# To Prevent Procreation by Confirmed Criminals, Idiots, Imbeciles and Rapists

**Published:** 1910-06

**Authors:** 


### To Prevent Procreation by Confirmed Criminals, Idiots, Imbeciles and Rapists.

To prevent procreation by confirmed criminals, idiots, imbeciles and rapists was the title of Senate bill No. 298, which failed of passage before the recent session of the Virginia Legislature. The bill was passed by the Senate, and was favorably reported by the House Committee, but defeated on the floor of the House after a close fight, much blind sentiment apparently figuring in the debate.

The corrective measure proposed seemingly being somewhat radical and unusual to the lay mind, it has evidently been hard for many to disassociate the idea that sterilization in the criminal was done for other purpose than that of punishment; consequently, with strong prejudices to overcome, such bill must await a better understanding of its true object, when, perforce, sentiment will give way to reason, and the prevention of procreation in selected cases will be demanded by law.—*Virginia Medical Semi-Monthly.*

